# Supplementary material for: Volume of hyperintense inflammation (VHI): A quantitative imaging biomarker of inflammation load in spondyloarthritis, enabled by human-machine cooperation
Source: PLoS One. 2023 Apr 19;18(4):e0284508. doi: 10.1371/journal.pone.0284508 (PMC10115260; doi:10.1371/journal.pone.0284508)
Supplement: S1 File — (DOCX) [file pone.0284508.s001.docx]

**Supplementary Information S1: Evaluation Metrics**

The similarity of a pair of binary segmentations was evaluated with the Dice coefficient, defined for the class of interest (abnormal or background) as the ratio of the number of pixels (voxels) having identical location in both segmentations to the average of number of pixels (voxels) in the segmentations [20]:

$$Dice= \frac{2|S_{1}\bigcap S_{2}|}{|S_{1}|+|S_{2}|}$$

where $S_{i,i\in\{1,2\}}$ represents a point set, containing pixel (voxel) coordinates, the subscript $i$ refers to the first or second segmentation and $S_{1}\bigcap S_{2}$ is the intersection of the sets. We refer to the Dice coefficient as the *area* or *volume overlap*, depending on whether two segmented areas or volumes were compared.

To evaluate a deep learning model performance during training, a soft, differentiable generalization of Dice was used, implemented as [21]:

$$Dice= \frac{2\sum_{i}^{N} r_{i}p_{i}}{\sum_{i}^{N} r_{i}^{2}+\sum_{i}^{N} p_{i}^{2}}$$

where summation runs over pixels of reference standard, $r_{i}\in R$ and network probability map, $p_{i}\in P$.
