# Supplementary figures and images for: Volume of hyperintense inflammation (VHI): A quantitative imaging biomarker of inflammation load in spondyloarthritis, enabled by human-machine cooperation
Source: PLoS One. 2023 Apr 19;18(4):e0284508. doi: 10.1371/journal.pone.0284508 (PMC10115260; doi:10.1371/journal.pone.0284508)

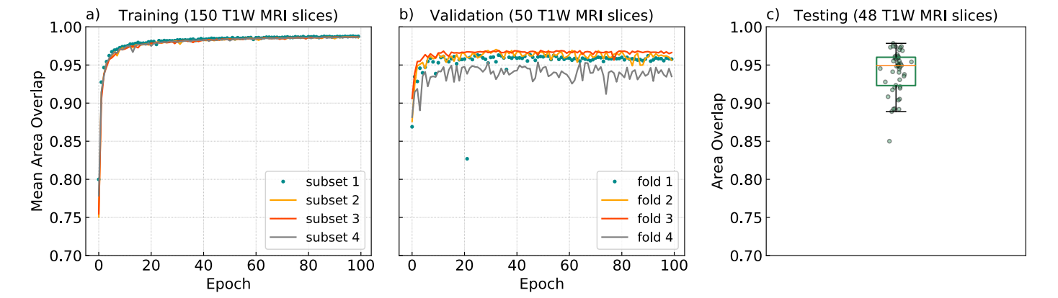

Supplement: S1 Fig — Mean area overlap (Dice score) vs training epoch for different training data subsets (a) and validation folds (b). Each point represents Dice score averaged over (i) classes (foreground & background), (ii) samples in a mini batch and (iii) 350 augmentation steps. Area overlap from pair-wise comparison of reference standard and rounded prediction on the test data from models averaging ensemble (three runs using all training data, 200 T1W image slices) (c). (TIF) [file pone.0284508.s002.tif]

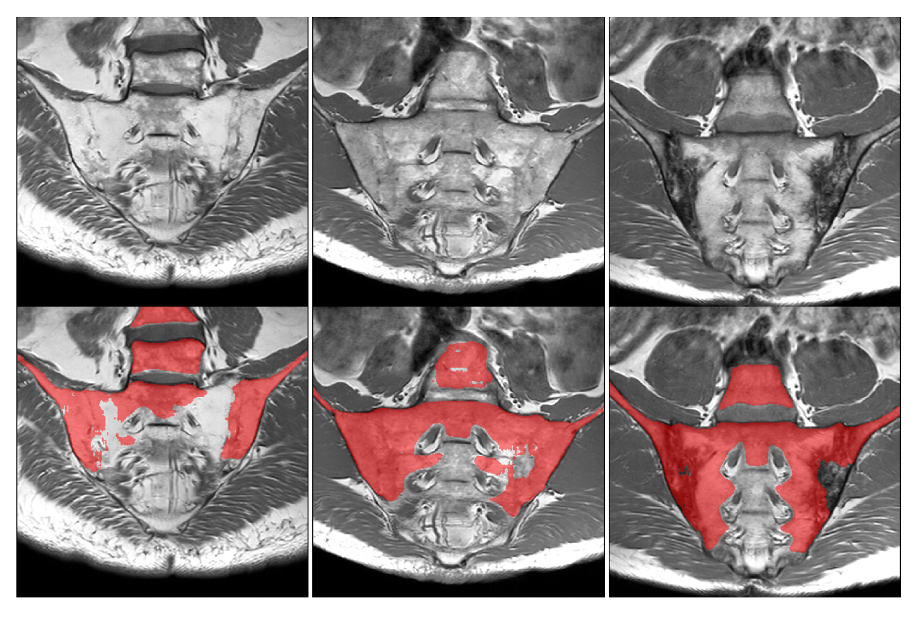

Supplement: S2 Fig — T1w image slices in oblique coronal plane (top) for three subjects with super-imposed models averaging ensemble rounded prediction (bottom). Subjects exhibit very abnormal bone, comprising either high fat content (left, middle) or sclerosis (right), leading to areas of ‘missing’ bone within the segmentations. (TIF) [file pone.0284508.s003.tif]

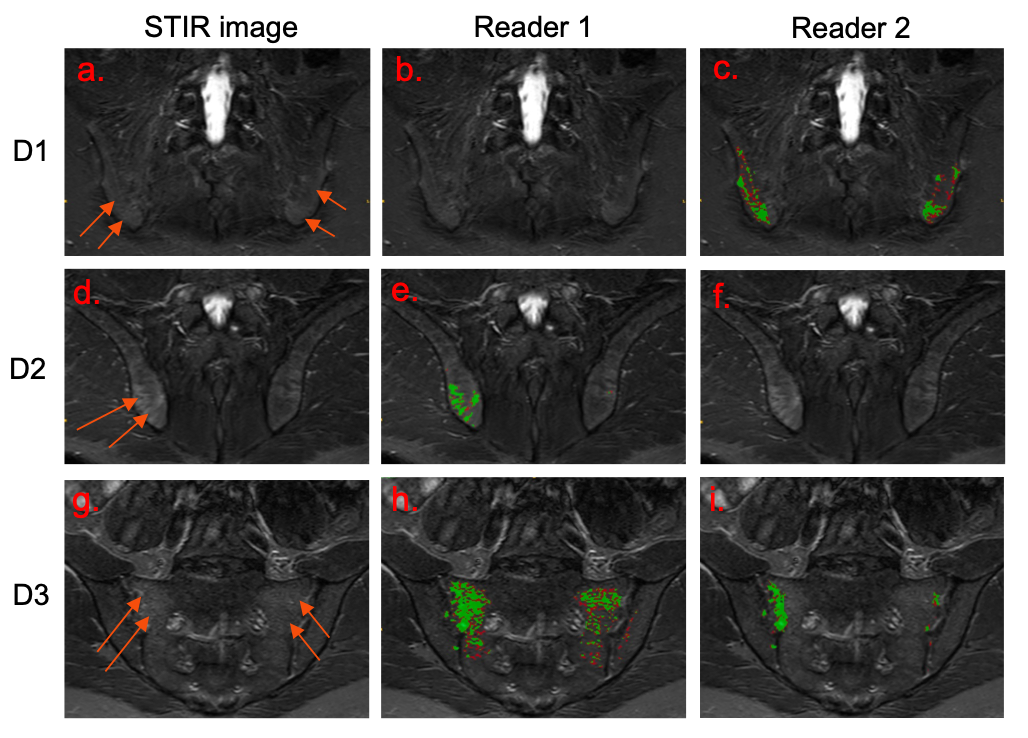

Supplement: S3 Fig — The three discrepancies are denoted D1-D3 and shown on separate rows; for each, the STIR image (left column) and segmentations for the two readers (middle and right column) are shown. The green and red segmentations correspond to the higher and lower segmentation thresholds. In two cases (D1, D2), the disagreement was ‘anatomical’ and related to the presence of hyperintensity in the posterior ilium, which can be attributed to either inflammation or variations in normal bone composition. In one case (D3) the disagreement was ‘artefactual’ and related to the presence of faint, diffuse hyperintensity in the potentially-inflamed subchondral bone region, which was deemed entirely inflammatory by one reader and partly artefactual by the other. (TIF) [file pone.0284508.s004.tif]
