# Supplementary material for: Volume of hyperintense inflammation (VHI): A quantitative imaging biomarker of inflammation load in spondyloarthritis, enabled by human-machine cooperation
Source: PLoS One. 2023 Apr 19;18(4):e0284508. doi: 10.1371/journal.pone.0284508 (PMC10115260; doi:10.1371/journal.pone.0284508)
Supplement: Attachment — Submitted filename: Response V3.docx [file pone.0284508.s005.docx]

Review Comments to the Author

Reviewer #1: The authors present a semi-automated workflow for quantitative imaging and diagnosis in STIR images. The workflow uses a deep neural-net (UNET) to segment regions of inflammation, while also relying on manual steps for image clean-up. The automatically identified regions of inflammation can then be used to create a quantitative assessment of the image, via volume of hyperintense inflammation (V_HI_), and potentially increase the diagnostic power, especially when compared to standard visual assessment approaches (e.g. SPARCC).

Overall, this was a well written paper with a grounded approach and corresponding discussion which underscored the potential role of AI-based image processing and quantitative imaging biomarkers in a clinical setting. The methods used in this study were sound and explained with adequate detail, and the various diagnostic criteria and scoring methodologies were clearly outlined for the reader. The discussion highlights the main conclusions of the various performance/comparison testing, and the Limitations section appropriately discusses the potential pitfalls of over-reaching conclusions from this limited study.

I recommend this study for publication pending revisions based on the comments/questions below.

**We thank the reviewer for their positive comments on the approach, study design, writing and manuscript structure.**

R1.1. Do all images go through step vi in the work flow (i.e. the manual cleaning process)? Or are only images which ‘fail’ segmentation put through manual cleaning? Could the authors please specify these details. How are images deemed ‘failures’?

**All images go through step vi in the workflow, but the extent to which the images are actually cleaned depends on the radiologist’s assessment of how much cleaning is required. This step is designed to maintain some radiological control over the pipeline, since this may increase confidence and flexibility compared to a purely automated workflow. We have clarified this point in the Methods section of the manuscript.**

**We would argue that the need for cleaning does not necessarily mean that the automated segmentation has failed. Rather, we expect some degree of ‘over-segmentation’ from the automated segmentation step, which intentionally priorities sensitivity over specificity. This makes the subsequent manual adjustment faster and easier, because adjusting a very sensitive segmentation (i.e. removing ‘chunks’ of oversegmented tissue) is dramatically faster and easier than correcting a very specific segmentation (which would require contouring).**

**Our proposed approach also allows the radiologist to exercise some judgement and may make the process more transparent and trustworthy than a fully-automated solution, and therefore more likely to be successfully implemented into clinical care. It also follows the important precedent set by coronary calcium scoring (see Refs 18-22 in the main article) as discussed in further detail below and in the manuscript itself.**

R1.2. Additional discussion of the necessity/time burden for the manual cleaning step of the workflow is warranted. Can this step also be automated? If so, what is the approach suggested by the authors? If not, how do the authors envision this manual step fitting into the workflow in a real clinical environment?

**Thanks, this is an important point and we have expanded the discussion of this point in the Discussion section, as suggested.**

**This last step in the pipeline could certainly also be automated but retaining a degree of manual input may be preferable to a fully automated solution. One problem with aiming for full automation is that a very large, high-quality training dataset - ideally incorporating data from multiple scanners - would likely be required to achieve satisfactory performance (and for the algorithm to be trusted for use in clinical care). We are not aware of a dataset of this type at present. Therefore, the proposed approach strikes a practical balance – it used deep learning to improve the precision of inflammation assessment but avoids the need for a very large training dataset.**

**Furthermore, we would highlight that a similar pipeline – that used for coronary calcium scoring (whereby an initial segmentation is generated automatically and then a radiologist adjusts this segmentation to retain only the relevant regions) – is already very widely used in clinical practice (see Refs 18-22 in the main article). As such, there is a clear precedent demonstrating that a cleaning step of this type can be feasible and practical in a real clinical workflow. This was one of the main motivations for our approach.**

**Nonetheless, there is potentially scope for further automation of this cleaning step, although we would approach this with caution in light of the need to maintain some radiological control, as discussed above.**

R1.3. The last discussion point in Section 4.1 reads: “… our results do indicate the subjective judgments made in the cleaning step of the workflow have a substantial impact on the Vhi measurements.” Unfortunately, this last point potentially undermines the claims in the study. Could the authors please estimate the impact or provide a performance comparison with and without the manual clean-up step?

**Thanks for this important point. We have considered this carefully and this has prompted us to adjust the abstract, introduction and discussion to better explain the rationale for a combined ‘human-machine’ approach. We think the main arguments for this are that (i) this is feasible without extremely large, carefully curated and labelled datasets (which are too costly to be practical for many fields, such as this) and (ii) in the absence of such a large dataset, a hybrid approach may be more trustworthy in clinical care than a purely automated approach. We have also provided further explanation of the relation of the work to recent literature on the subject of human / machine collaboration.**

**On the point about the impact of cleaning adjustments – we agree that this is important, although it should not detract from the fact that agreement between observers is markedly improved with the semiautomated method compared to purely manual segmentation.**

**The proposed performance assessment is already present – if no cleaning is performed the agreement between observers would be perfect (since the automated step does not vary), so the agreement statistics as stated give a direct indication of the impact of this cleaning step.**

**Note that the final radiologist-cleaned segmentations also show much closer agreement with each other (Dice 0.84) than they each do with the preliminary segmentation (Dice 0.60 / 0.55) for Reader 1 / Reader 2, indicating the importance of this step.**

R1.4. Could the authors also include in the Discussion how they envision creating future data sets to improve their algorithm? Importantly, what is the best way to define the ‘gold standard’ segmentation or diagnosis? Should machine learning approaches be using consensus results from trained physicians, or is the current approach adequate?

**Yes, this is an important point and one which we have spent some time considering, although in our opinion there may not be a single correct answer to this question.** **We have expanded the discussion of this point in the text.**

**Ideally, we would use a consensus of a substantial number of radiologists to generate the reference standard against which algorithms could be evaluated. This dataset would incorporate data from multiple scanners (with different acquisition protocols), thus enabling assessment of the generalisability of different methods. Algorithms with varying degrees of automation could then be evaluated against this reference standard in terms of both accuracy and precision (in the context of the differing clinical relevance of different inflammation loads).**

**This comparison is beyond the scope of the present work as a large multisite, multiscanner, multireader dataset is not currently available to us; however, this work lays the foundations for a future study of this type.**

R1.5. Further discussion in section 4.1 on the limitations of the defined ‘gold standard’ is warranted. Are there better approaches to creating a standard image, i.e. through consensus of board-certified clinicians?

**We have expanded the Discussion on the limitations of the reference standard in the text. Please also see our Response above on potentially better approaches to creating a reference standard.**

R1.6. In the discussion, 2nd paragraph: “ comparison on the non-transformed data…” Could the authors please specify the ‘transformation’ they are referring to? Is this purely the log transformation?

**Yes – this is the log-transformed data. We have now stated this explicitly in the location referred to.**

R1.7. Figure 5 caption should define R1 and R2.

**Thanks, this has been added.**

R1.8. Figure 7, the bottom left-most plot appears to have mislabeled axes. Should the axes be the difference between scores, not a comparison between readers? Additionally, I recommend labeling these plots a, b, c etc. to help guide the reader.

**Thanks for pointing out this error, which we have corrected. We have also added labels to the subplots.**

R1.9. Figure 9 should be referenced in section 2.7.3

**Thanks, we have referred to Figure 9 in this section.**

Reviewer #2: In this article, Hepburn et al present a workflow to analyze MRI images that enables precise quantification of V_HI_ (volume of hypertense inflammation), through a combination of a CNN, intensity based thresholding, and a manual cleaning step. This work compares the performance of the semiautomated image analysis pipeline with manual SPARCC scoring, and convincingly shows that the presented approach is able to extract the VHI from STIR sequences. By analyzing a single image, segmentation can be propagated to the remainder slices, thus simplifying VHI extraction in comparison to individual image segmentation. By defining a ‘normal bone’ region, the workflow enables segmentation of MRI images that differ in intensities, textures, and plausibly, acquisition settings. This work highlights the usefulness of CNNs and computer vision not only to facilitate image diagnostics, but also to make the process more rigorous and accurate. It appears that VHI is not a common metric to assess disease state or clinical outcomes, and thus this work aims to both develop a pipeline for accurate VHI quantification, as well as propose VHI as a feature to be relied on as a clinically-relevant metric. The latter is not fully validated in this work. However, the presented work, although not fully optimized, nicely shows how computer vision can be harnessed for medical diagnostics. I believe this work would be of interest to a broad community. I have a few items I believe should be addressed prior to publication:

**We thank the reviewer for their careful appraisal of our article and their positive comments on our approach and article.**

R2.1. Can the authors provide some background for non-experts on the rationale or background to use VHI as a biomarker for spondyloarthritis?

**Yes, we have expanded the discussion of the relevant previous literature (particularly around the pre-existing qualitative scoring methods, such as the SPARCC score) in the Introduction section so that the reader can better understand the rationale for using this biomarker.**

R2.2. Can the authors comment on whether the pipeline would be robust to MRIS acquired with different acquisition settings?

**This work used an aggressive augmentation strategy in order to improve robustness between scans; this should also make the scans more robust to different acquisition settings. However, it would clearly also be desirable to train on a larger training dataset, ideally involving data from multiple scanners, in order to further improve the robustness of the network.**

**Although AI-based methods typically need to be trained on multiple types of scanner (and multiple acquisition settings) in order to be generalisable, we would expect that our approach (whereby the task set for the AI – i.e. segmentation of normal bone – is relatively simple) could be more generalisable than methods which are directly trained on areas of pathology (since this is a much more challenging task).**

**We have expanded the discussion of this point in the manuscript.**

R2.3. It would appear that the lower intensity limit (L_lower) can be simply calculated by knowing the value of IQR. The need for an iterative search is unclear.

**Thanks, this is true in cases where the difference between the initial estimate of L_lower_ and the value of L_upper_ greater than half the IQR and therefore adjustment is required (alternatively, if the difference between difference between the initial estimate of L_lower_ and the value of L_upper_ is less than half the IQR, then L_lower_ = QU + n.IQR). We only implemented this as an incrementation to dictate that the values of n were discrete, which enable us to simply track the value for different subjects, although we agree that the suggested approach would have been conceptually simpler. In practical terms the two options are essentially identical.**

R2.4. How common is it for patients to have ‘abnormal bone marrow’, that would render the pipeline unusable?

**In our experience this is relatively rare (3/29 subjects in our dataset); the main problem was that these cases were not present in our training set. We would expect that training on a larger dataset would reduce or eliminate this problem. This issue has already been mentioned in the Discussion.**

R2.5. Fig 4 and 5 seem a bit redundant. If there is a standard reference that contains information from multiple readers, but the readers don’t tend to agree. This point could suggest the standard reference is not as reliable as possible. Could a third observer be included?

**Thanks, this is a fair point. Figure 5 was simply included as an additional way of providing some intuition for the reader, but on reflection we agree with the reviewer and have removed this. In its place, we included an alternative figure showing that the combined human-machine workflow provides accurate measurements with respect to pure manual segmentation, providing a more transparent analysis against an independent reference standard.**

R2.6. Although it is clear that the relationship between VHI and SPARCC is improved with a logarithmic transformation, it is unclear why this is so, or why this is justifiable. Can the authors expand on this choice?

**The logarithmic transformation is simply intended to improve the visibility of the points, since there are otherwise (with a standard linear scale) many points clustered at low V_HI_ values which overlap and therefore cannot be seen clearly. By showing both the standard linear scale and log-transformed data, we aim to display the data as fully as possible so that no datapoints are ‘hidden’ from the reader by this phenomenon. In other words, the use of the log(x+1) transformation allows the behaviour of low V_HI_ values to be appreciated as well as the behaviour of the high values.**

**None of our conclusions are contingent on this transformation, and we have highlighted in the Discussion that the two different scales may enable visualisation of different aspects of the V_HI_ behaviour.**

R2.7. Fig 9: linear relationship between VHI and SPARCC change, I do not think the slope or intercept values are as informative as the fit, can the authors provide the R2 as well?

**Yes, we have added this, although we do not feel it provides substantial additional information to the way that the data are interpreted. The relationship between SPARCC change and V_HI_ change is relatively weak, probably because the two metrics provide somewhat different information on response. We take the view that the plot itself is probably the most useful way of presenting the data.**

R2.8. The failure analysis lacks evidence. Determining why two of the samples were deemed ‘anatomical’ failures and the other one was ‘artefactual’ is not clear. This could be because it requires expertise on this type of images, in which case further explanation is warranted. If there is no data to support these claims, I suggest they are left to the reader’s interpretation. In addition, what is the significance of failures being anatomical or artefactual?

**We apologise for not explaining this more clearly in the original submission.**

**These are radiological judgements (i.e. they do require an appropriate level of expertise) and are designed to provide a more granular explanation of the reasons for failure; they also fulfil item 37 of the CLAIM checklist (which advises that particular examples of incorrectly-classified cases should be highlighted and discussed). We have provided further detail on the classification scheme in the Methods section to allow the reader to better understand this classification.**

R2.9. Is there anything potentially useful included in SPARCC scoring that VHI does not take into account?

**Overall, aside from AI errors, it seems unlikely that the SPARCC score contains useful information that would not be included in V_HI_. Although the SPARCC score does contain additional binary scores for particularly deep or bright information, we would argue that these are essentially surrogates which provide additional dynamic range to the SPARCC score (which only provides binary classifications in each joint quadrant), allowing it to capture a higher overall burden of disease. By comparison, V_HI_ is measured on a continuous scale and does not suffer from the same problem with dynamic range; this is reflected in the approximately exponential relationship between SPARCC and V_HI_, which suggests that V_HI_ is much better able to capture large burdens of disease.**

Minor comments

R2.10. I recommend defining acronyms, the manuscript is loaded with MRI / radiology jargon and acronyms and can be hard to follow.

**Thanks, we have ensured that acronyms that are defined at first use and have attempted to simplify the language where possible.**

R2.11. In 3.3, is this referring to VHI or disease region?

**3.3 refers to the final disease segmentation (i.e. the cleaned output at the end of the pipeline) – we have specified this in the title of this subsection.**

R2.12. When classifying patients as responders / non-responders, how was this performed when using VHI or SPARCC scoring? Was a specific % change deemed necessary? If so, how was this determined?

**This is detailed in Section 2.7.3. We have included the text here for reference:**

*‘Changes in V_HI_ and visual scores after treatment were visualized using spaghetti plots in which changes in both measurements for individual patients were depicted (Figure 9). Separate plots were generated for those patients who showed evidence of response to therapy using clinical criteria and for those patients who did not. To provide a numerical summary of the ability of V_HI_ to capture response, we recorded the agreement between clinical response assessment and V_HI_-based response assessment and between clinical response assessment and SPARCC-based response assessment. For the purposes of this analysis, any patient undergoing an improvement in V_HI_/SPARCC was deemed to be a V_HI_/SPARCC responder. Note that this is an imperfect assessment and an alternative would be to have a threshold for response based on the variance in the data, however, the latter is problematic when the distributions of the data are so different for V_HI_ and SPARCC and risks creating an unfair threshold depending on the specific transformation used. The proposed approach, whereby any improvement is regarded as a response, is not regarded as a clinically meaningful threshold but rather as a useful simplification for the purpose of this analysis.’*

R2.13. Fig 4c. I believe the caption is incorrect

**We have reviewed this caption carefully and, apart from a small typo, cannot identify an error. We would be happy this if there is something specific that the reviewer has in mind.**
